# Metabolomic profiling of renal cyst fluid in advanced ADPKD: insights from dialysis and transplantation cohorts

**DOI:** 10.1007/s11306-025-02291-7

**Published:** 2025-06-26

**Authors:** Simon Heckscher, Nicolas A. Ihlo, Jan Schueler, Fabian Kellermeier, Jens M. Werner, Barbara Nuebel, Verena Gross, Matthias May, Bernd Wullich, Martin Kammerl, Carsten Gnewuch, Ralph Burkhardt, Björn Buchholz, Eric Pion, Thiha Aung, Miriam Banas, Hans J. Schlitt, Peter J. Oefner, Katja Dettmer, Wolfram Gronwald, Merle Behr, Silke Haerteis, Katharina M. Schmidt

**Affiliations:** 1 Institute of Functional Genomics, University of Regensburg, Regensburg, Germany; 2 Faculty of Informatics and Data Science, University of Regensburg, Regensburg, Germany; 3 Institute for Molecular and Cellular Anatomy, University of Regensburg, Regensburg, Germany; 4 Department of Surgery, University Hospital Regensburg, 93053 Regensburg, Germany; 5 Department of Urology and Pediatric Urology, University Hospital Erlangen, Erlangen, Germany; 6 Department of Urology, St. Elisabeth Hospital Straubing, Brothers of Mercy Hospital, Straubing, Germany; 7 Faculty of Applied Healthcare Science, Deggendorf Institute of Technology, Deggendorf, Germany; 8 Institute for Clinical Chemistry and Laboratory Medicine, University Hospital Regensburg, Regensburg, Germany; 9 Department of Nephrology and Hypertension, University Hospital Erlangen, Erlangen, Germany; 10 Department of Nephrology, University Hospital Regensburg, Regensburg, Germany

**Keywords:** ADPKD, Cyst fluid, Metabolomics, NMR spectroscopy, Mass spectrometry, Patient clustering

## Abstract

**Background:**

Autosomal dominant polycystic kidney disease (ADPKD) is the most common hereditary kidney disorder characterized by progressive renal cyst formation, often leading to end-stage kidney disease (ESKD). In contrast to the urinary metabolome in ADPKD, the composition of renal cyst fluid remains largely unexplored.

**Methods:**

We conducted a comprehensive metabolomic analysis of renal cyst fluid from 26 ADPKD patients (20 on dialysis, six with kidney transplants) using ¹H-NMR spectroscopy and liquid chromatography-mass spectrometry (LC-MS). Cysts were clustered based on metabolite profiles, and differences were analyzed across groups defined by renal function status (dialysis vs. transplant), cyst volume, and cyst fluid sodium concentrations.

**Results:**

Dialysis patients and transplant recipients differed significantly in their renal cyst fluid metabolomes. The former exhibited higher concentrations of myoinositol, creatinine, sucrose, τ-methylhistidine, trigonelline, and sarcosine, while the latter showed increased levels of leucine, isoleucine, valine and alanine. Remarkably, metabolites of the immunosuppressive prodrug mycophenolate mofetil were detected in renal cyst fluids after kidney transplantation. Despite intra- and interindividual variability, cyst fluid from the same patient displayed greater homogeneity. Interestingly, metabolomic profiles were not altered by cyst size.

**Conclusion:**

This first systematic metabolomic analysis of renal cyst fluid in advanced ADPKD reveals distinct metabolic signatures linked to renal function status. The data provides novel insights into the pathophysiology of ADPKD and highlight the potentials of renal cyst fluid metabolomics for identifying biomarkers and therapeutic targets.

**Supplementary Information:**

The online version contains supplementary material available at 10.1007/s11306-025-02291-7.

## Background

Autosomal dominant polycystic kidney disease (ADPKD), one of the most frequent human monogenic disorders and the fourth leading cause for kidney failure worldwide, is characterized by the progressive development of numerous fluid-filled cysts within the renal parenchyma (Chapman et al., 2015; Harris & Torres, 2009). ADPKD is predominantly caused by mutations in the PKD1 or PKD2 gene encoding for polycystin-1 and polycystin-2. Both proteins are integral to the function of renal tubular epithelial cells and the maintenance of normal tubular architecture and function (Torres et al., 2007a; Wilson, 2004). In ADPKD, formation and expansion of cysts are both driven by a complex interplay of genetic, molecular, and cellular mechanisms. Cyst initiation often starts early in life and involves focal dedifferentiation and proliferation of tubular epithelial cells derived from nephrons and collecting tubules, followed by fluid secretion into the cyst lumen (Grantham et al., 2011; Paul & Vanden Heuvel, 2014; Torres et al., 2007a). Cyst formation and growth are accompanied by inflammation and fibrosis of the surrounding parenchyma (Fragiadaki et al., 2020). Common clinical symptoms of ADPKD are abdominal pain, urinary tract infections, hematuria, hypertension, and eventually renal failure (Cornec-Le Gall et al., 2019; Gaur et al., 2019). Half of patients reach end-stage kidney disease (ESKD) by age 60 requiring some method of renal replacement therapy. Hemodialysis and peritoneal dialysis are both appropriate options, kidney transplantation remains the preferred choice if possible (Al Khunaizi & Alam, 2018).

A key aspect of ADPKD pathology is the complex composition of the renal cyst fluid and its paracrine cystogenic effects. Cyst fluid contains more than 350 unique proteins and other biochemically active substances (Kenter et al., 2019; Lai et al., 2008), including products of systemic and local metabolism. These molecules may play critical roles in cyst growth, maintenance and structural formation, and disease progression (Kenter et al., 2019; Ye et al., 1992; Yamaguchi et al., 1995). During the early stages of ADPKD, cysts are attached to their parental renal tubule. Beyond a diameter of 2 mm, cysts close off. Therefore, cyst fluid initially is derived from the glomerular filtrate, and in later stages from a combination of plasma ultrafiltration and secretory activity of the cyst-lining epithelium (Grantham et al., 2011; Paul & Vanden Heuvel, 2014; Sullivan et al., 1998; Terryn et al., 2011).

Understanding the detailed metabolomic landscape of renal cyst fluid and its interactions with cyst epithelial cells may offer valuable insights into potential therapeutic strategies for ADPKD. While the urinary metabolome in ADPKD has been extensively studied (Dekker et al., 2020, 2022; Gronwald et al., 2011; Hallows et al., 2023; Houske et al., 2023), this is the first systematic analysis of renal cyst fluid in advanced-stage ADPKD post-nephrectomy, including 20 dialysis patients and six kidney transplant recipients. Employing proton nuclear magnetic resonance (^1^H-NMR) spectroscopy and ultra-performance liquid chromatography (UPLC) hyphenated to a quadrupole time-of-flight mass spectrometry (qTOFMS) system together with state-of-the-art data analysis, we seek to gain new insights into the potential pathophysiological processes relevant for ADPKD progression.

## Methods

### Patient selection and sampling of cystic fluid

The study included ADPKD patients with ESKD that underwent nephrectomy at the University Hospital Regensburg, Germany, from October 2020 until May 2024, at the University Hospital Erlangen, Germany, from February 2024 until May 2024, or at the St. Elisabeth Hospital Straubing, Germany, from October 2023 until May 2024. After exclusion of seven patients due to macroscopic contamination of cyst fluid with blood or pus, 26 participants remained: 20 on dialysis and six with kidney transplants.

Immediately after nephrectomy, renal cystic fluid was obtained from cysts of various size (0.5-1 mL, 1–3 mL, > 3 mL), centrifuged and stored at -80 °C for further analysis.

### ^1^H-NMR spectroscopy, LC-MS, and sodium measurements

^***1***^***H-NMR:*** NMR experiments were performed as described previously (Gronwald et al., 2008). In short, 400 µL of cyst fluid were mixed with 200 µL of 0.1 M phosphate buffer (pH 7.4), 50 µl of 0.75% (w) TSP-2,2,3,3-d4 in deuterium oxide (Sigma-Aldrich, Taufkirchen, Germany) and 10 mL of a 240 mM stock solution of formic acid, which served as an additional internal standard that, unlike TSP, does not bind to protein. Spectra were acquired in 5 mm tubes on a 600 MHz Bruker Avance III spectrometer, using a triple resonance (^1^H, ^13^C, ^15^N, ^2^H lock) helium cooled cryoprobe with z-gradient. Handling of samples was done by an automatic Bruker SampleJet sample changer (Bruker Biospin GmbH, Ettlingen, Germany). Tuning and matching of the probe as well as locking and shimming of the sample were performed automatically. One-dimensional ^1^H NMR spectra were obtained at 298 K using a Carr-Purcell-Meiboom-Gill (CPMG) pulse-sequence with solvent signal suppression by presaturation during relaxation time. Spectra were automatically Fourier transformed and phase and baseline corrected. For quantification of metabolites, NMR spectra were analyzed by Chenomx NMR© software suite v. 9.0 (Chenomx Inc., Edmonton, Canada). Signal assignments were additionally verified by spike-in experiments and a high-resolution 2D ^1^H-^13^C HSQC spectrum that was matched with the corresponding 2D pure compound reference spectra of the Bruker BBIOREFCODE database (Bruker BioSpin GmbH, Ettlingen, Germany).

***LC-MS:*** For protein precipitation, cyst fluid samples were extracted with 80% methanol. Untargeted metabolomics was performed using a Thermo Scientific Dionex Ultimate 3000 UPLC system (Idstein, Germany) coupled to a SCIEX Triple TOF 5600 + mass spectrometer (Sciex, Framingham, Massachusetts, USA). Chromatographic separation was carried out using an ACQUITY Premier HSS T3 reversed phase column with VanGuard FIT (Waters, 1.8 μm, 2.1 mm x 150 mm). For data evaluation, raw data files were converted into.mzml-file format using the peak picking function in MSConvert (version 3.0.19, ProteoWizard, Palo Alto, CA, USA) with the parameter “vendor” (Chambers et al., 2012). Untargeted data processing with peak detection, deconvolution, peak alignment, and gap filling was performed with MZmine2 version 2.53 (Pluskal et al., 2010). A detailed description of the method is given in the supplementary material.

***Sodium measurements:*** Sodium measurements in ADPKD cyst fluid were performed at the Institute of Clinical Chemistry and Laboratory Medicine at the University Hospital Regensburg using an indirect ion-selective electrode method on a cobas^®^ pro analyzer from Roche Diagnostics GmbH (Mannheim, Germany). If the original cyst fluid volume was insufficient due to the small size of the cysts, specimens from the ^1^H-NMR measurements were used. An aliquot of 340 µL of water was added to all ^1^H-NMR measurement samples to achieve a final volume of 1 mL.

### Data analysis

Quantitative NMR and MS-fingerprinting data were PQN-normalized (Dieterle et al., 2006) to account for unwanted technical and biological variance. The MS-fingerprinting data was additionally log-scaled. For clustering, missing values in the NMR dataset were imputed using the lowest concentration determined for that molecule and all measurements were standardized using z-scores. The resulting data was hierarchically clustered for both samples and features by performing average linkage according to their cosine distances using SciPy (Virtanen et al., 2020). By using these linkages, the data was visualized through the clustermap function of Seaborn (Waskom, 2021). Additionally, dimension reduction and visualization with UMAP (McInnes & Healy, 2018) was performed.

We considered three groupings of the cyst samples to detect differences in fluid composition, namely patients’ renal function (either requiring dialysis or having a functioning renal allograft), volume of the cyst (small: volume < 1 mL, medium: 1–3 mL, or large: > 3 mL), and sodium level of the cyst fluid (low: < 100 mmol/L or high: > 100 mmol/L). For the grouping based on renal function, differences in molecule measurements were tested using the Mann-Whitney U rank tests. Here we considered for each patient the median of the measurements from their cysts and assumed sample independence. Additionally, properties of the patients, grouped according to renal function, were compared using the Mann-Whitney U rank test if numerical and Fisher’s exact test if binary. For these tests, the implementations in SciPy (Virtanen et al., 2020) were used.

For the comparisons of molecules based on volume and sodium level of the cysts, a Linear Mixed Effects Model was used to account for differences between patients, using MixedLM from Python package statsmodels (Seabold & Perktold, 2010).

## Results

### Patient characteristics

26 advanced-stage ADPKD patients who underwent a nephrectomy were included. 20 patients were currently on renal dialysis and six had received a kidney allograft 12 to 60 months ago subsequent to years on dialysis. The mean age of the entire cohort at ADPKD diagnosis was 25.8 ± 8.2 years. Mean age at first dialysis and time of nephrectomy was 47.8 ± 8.9 years and 53.8 ± 8.0 years, respectively. In most cases, nephrectomy was performed due to a lack of space before kidney transplantation. Other indications for nephrectomy comprised abdominal pain and indigestion, recurrent infections, hematuria/hemorrhage, and suspicion of malignancy. Weight of removed organs ranged from 779 up to 5,800 g. Detailed patient characteristics are summarized in Table 1, their medication in Supplementary Table 2.

**Table 1:** Patient characteristics of the ADPKD cohort

		All	Dialysis	Transplant	*p*-value
N (percentage)		26 (100%)	20 (76.9%)	6 (23.1%)	•
Age	Years	53.8 ± 8.0	53.1 ± 8.3	56.0 ± 7.3	0.4648
Sex	m = 1, f = 0	18 (69.2%)	13 (65.0%)	5 (83.3%)	0.6279
Age at time of diagnosis	Years	25.8 ± 8.2*	26.0 ± 8.4*	25.0 ± 8.2	0.6883*
Age at time of first dialysis	Years	47.7 ± 8.9	47.3 ± 9.4	49.2 ± 7.4	0.7139
Previous contralateral nephrectomy	0/1	8 (30.8%)	3 (15.0%)	5 (83.3%)	0.0045
Serum creatinine	mg/dl	8.4 ± 5.3	10.5 ± 4.2	1.5 ± 0.6	0.0003
Dialysis at date of nephrectomy	0/1	20 (76.9%)	20 (100.0%)	0 (0.0%)	0.0000
Kidney transplant recipient	0/1	6 (23.1%)	0 (0.0%)	6 (100.0%)	0.0000
Time since transplantation	Months	•	•	32.3 ± 19	•
Weight of removed kidney	g	2439 ± 1331	2646 ± 1452	1818 ± 586	0.3670
Indication of nephrectomy					
Less space for transplant	0/1	15 (57.7%)	15 (75.0%)	0 (0.0%)	0.0020
Abdominal pain, indigestion	0/1	10 (38.5%)	8 (40.0%)	2 (33.3%)	1.0000
Recurrent infections	0/1	2 (7.7%)	1 (5.0%)	1 (16.7%)	0.4154
Hematuria, hemorrhage	0/1	2 (7.7%)	2 (10.0%)	0 (0.0%)	1.0000
Suspicious for malignancy	0/1	3 (11.5%)	0 (0.0%)	3 (50.0%)	0.0077

Given are the absolute number and percentage or mean value and standard deviation. The last column contains the p-value as calculated using the Mann-Whitney U rank test for numerical data and Fisher’s exact test for binary data. * Age at time of diagnosis could not be evaluated in two cases, so mean value, standard deviation and p-value were calculated using the remaining 24 cases.

### Metabolite composition of renal cystic fluid

**Fig. 1:**
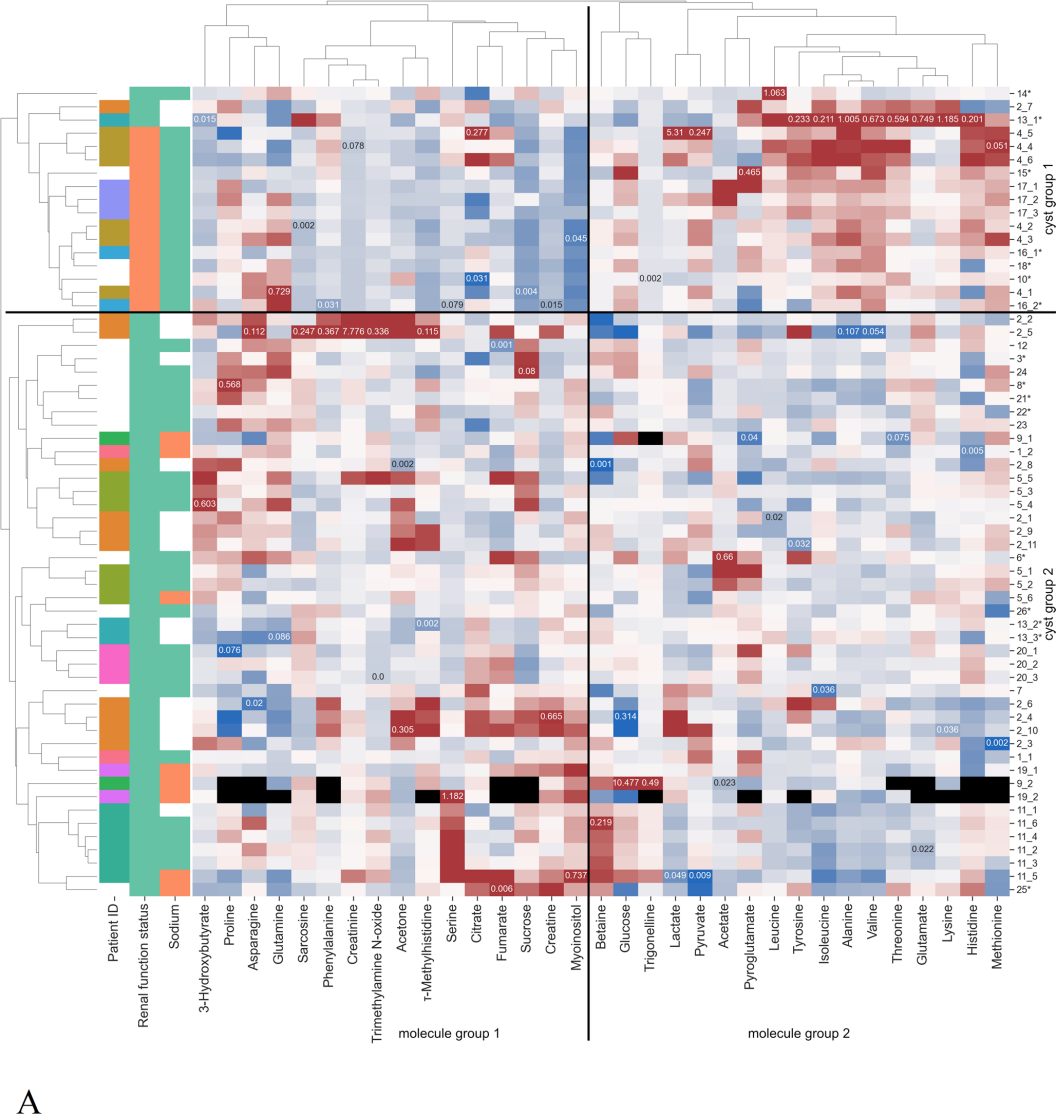

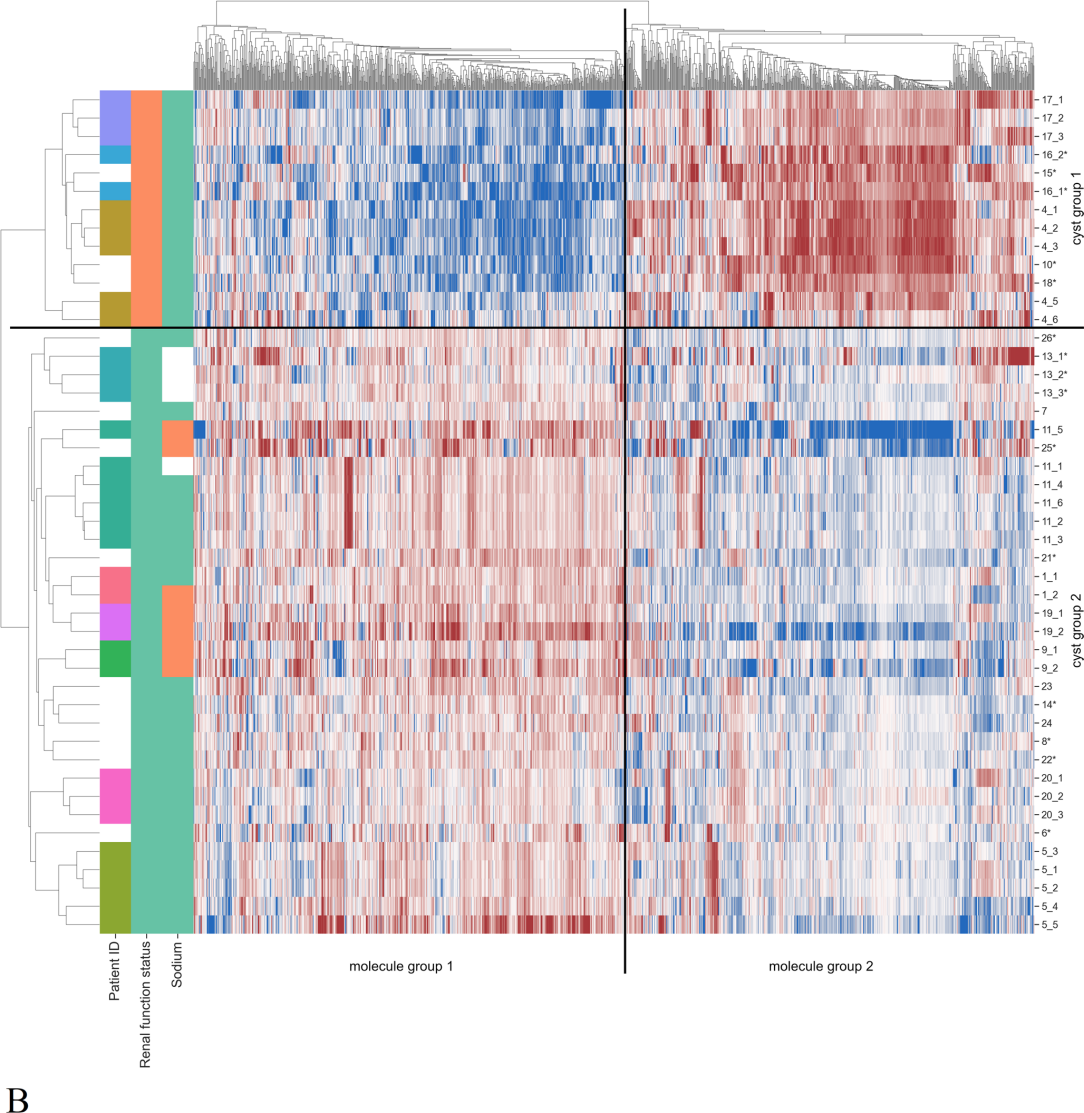
Clustered heatmaps of cyst fluid measurements. **A** Absolute quantified ^1^H-NMR and **B** MS fingerprinting data. First column indicates attribution of cysts to patients with white entries for patients with only one cyst sampled. Second column shows the renal function status: dialysis (green) or transplant (orange). Third column depicts the cyst sodium concentrations with values above (green) or below (orange) 100 mmol/L, non-reliable sodium measurements are left white. Larger and smaller values are depicted in red and blue, respectively, while missing values are shown in black. In Figure **A**, the highest and lowest measured concentrations (mM) for each molecule are given in the respective cells. Groups of patients and molecules based on the clustering are also marked. The rows are marked by the cyst ID with * indicating fluid which may be a mixture from multiple cysts

To analyze the metabolite composition of renal cystic fluid, ^1^H-NMR spectroscopy (in total 61 cysts from 26 patients) and LC-MS (in total 46 cysts from 23 patients) were conducted. Numbers of specimens ranged from one to 11 per individual. Figure 1 shows the results of both measurement methods as clustered heatmaps. Note that Fig. 1A displays metabolites quantified by NMR, while Fig. 1B gives a broad metabolic overview provided by MS-fingerprinting data.

Clustering revealed two groups of cysts with respect to their metabolomic profile, in both NMR and MS data. These groups mostly align with the renal function status of the respective patients. Thus, we will be considering the cysts from transplant patients (called “transplant group”) and dialysis patients (“dialysis group”) in their place from now on. Also, the molecules appear to form two clusters, which are highlighted as “molecule group 1” and “molecule group 2”. From Fig. 1 it becomes apparent that cyst fluids of the dialysis group tend to reveal higher values for molecules in group 1, and lower values for molecules in group 2, whereas this trend is reversed in the transplant group.

For NMR data, testing for differences between the dialysis and the transplant group identified ten metabolites that differed significantly in distribution (see Supplementary Fig. S1). Six of those compounds trended to have higher concentrations in cysts from dialysis than transplant patients. Those are myoinositol (Bonferroni-corrected p-value 2.9 × 10^− 4^), creatinine (2.9 × 10^− 4^), sucrose (5.7 × 10^− 4^), τ-methylhistidine (8.6 × 10^− 3^), trigonelline (1.8 × 10^− 2^), and sarcosine (3.6 × 10^− 2^). Their concentrations also vary more within the dialysis group than in the transplant group (see Supplementary Fig. S1). Except trigonelline, these molecules belong to “molecule group 1”, exemplifying the trend observed before. In the transplant group, higher concentrations were detected for alanine (p-value 2.9 × 10^− 4^), isoleucine (3.4 × 10^− 3^), valine (9.7 × 10^− 3^), and leucine (4.8 × 10^− 2^). These belong to “molecule group 2”, together with 6 other proteinogenic amino acids, while only 5 proteinogenic amino acids belong to “molecule group 1”. Note that the signal assignment of the significantly regulated metabolites was additionally verified by spike-in experiments.

Based on the MS data, 364 features were significantly different between the transplant and the dialysis group as illustrated by the volcano plot in Supplementary Fig. S2. Significantly different features with p-value, log2 fold change, exact mass, retention time and partial identification with indication of the level of the ID are listed in Supplementary Table 1. In accordance with the NMR data, isoleucine and leucine were significantly higher in the transplant group. Among the ten features with the highest absolute fold change, two features could be assigned to glucuronides of mycophenolic acid. The signal at 7.6 min was identified as mycophenolic acid glucuronide (MPAG) using an authentic standard. The signal at 6.59 min was putatively assigned to mycophenolic acid acyl glucuronide (AcMPAG). Mycophenolic acid is an immunosuppressant derived from the prodrug mycophenolate mofetil that was given to all six transplant patients. Accordingly, its metabolites were only found in the transplant group. For data analysis, zero values present in the dialysis group were replaced by small imputed values and therefore the obtained p-values and fold changes for these two metabolites have to be treated with care. Mycophenolic acid undergoes phase 2 conjugation by the uridine diphosphate glucuronosyl transferase and is readily excreted to over 90% into the urine by glomerular filtration and active tubular secretion, predominantly as MPAG (Bullingham et al., 1998).

### Intraindividual and interindividual differences of cyst fluid composition

**Fig. 2:**
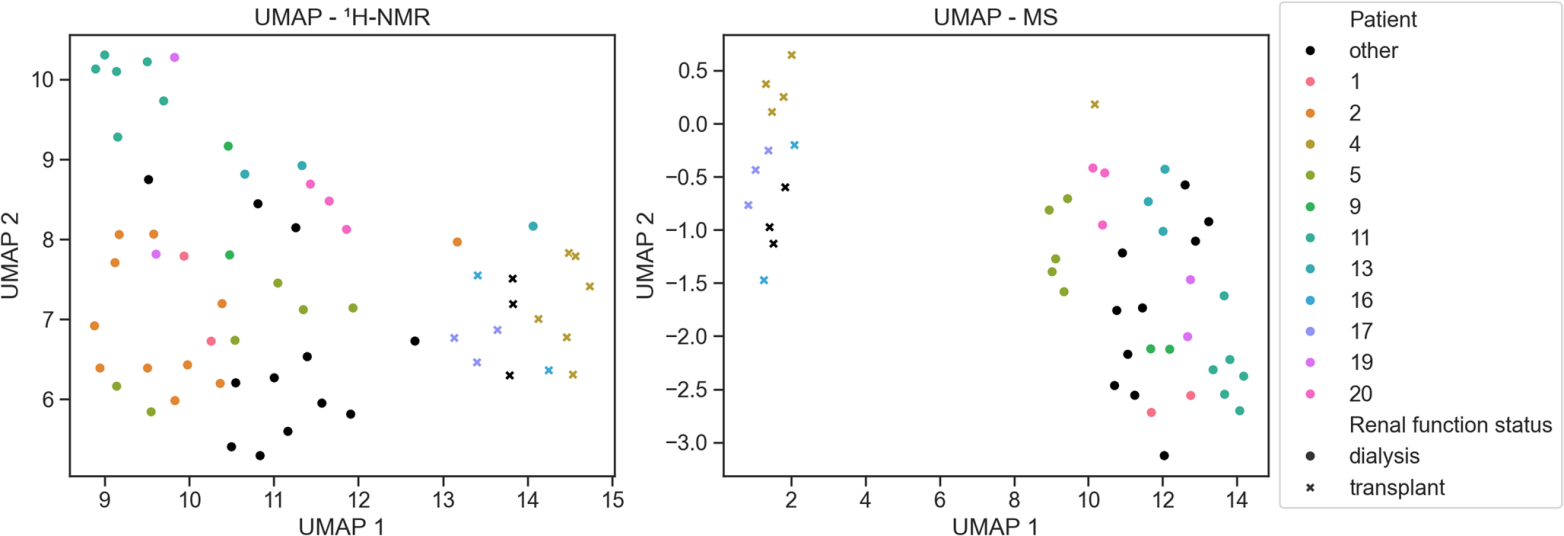
UMAPs of ^1^H-NMR (left) and LC-MS (right) data. Measurements of different cyst fluid samples from the same patient are depicted by the same color. Patients that had only a single cyst sampled and analyzed are shown in black. Renal function status is marked by dots (dialysis) and crosses (transplant).

Both clustering (see Fig. 1) and UMAP dimension reduction (see Fig. 2) demonstrate that different cyst fluid samples from the same patient are more similar than cyst fluid samples from different patients. This is exemplified by patient 11 (dark green in Figs. 1 and 2), whose samples form a tight cluster with no overlap with other patient samples in that group (see Fig. 2: ^1^H-NMR: top left corner, LC-MS: bottom right corner).

Additionally, UMAP visualization (see Fig. 2) underscores the dichotomy of the cyst metabolome of dialysis patients (left for ^1^H-NMR, right for MS) and transplant recipients (right for ^1^H-NMR, left for MS), already described before.

### Impact of cyst volume on cyst fluid composition

Differences between cysts of different volumes (45 cysts from 13 patients (11 dialysis, 2 transplant)) were tested for both, NMR and MS measurements, using a Mixed Linear Effects Model. NMR measurements yielded a single significant metabolite, namely sarcosine (Bonferroni corrected p-value of 2.4 × 10^− 4^), but this difference was driven mainly by one measurement for patient 2. The effect of cyst volume on sarcosine is not significant when excluding this measurement. Among the MS data, no molecules revealed a significant difference.

### Sodium concentrations of cyst fluid

**Fig. 3:**
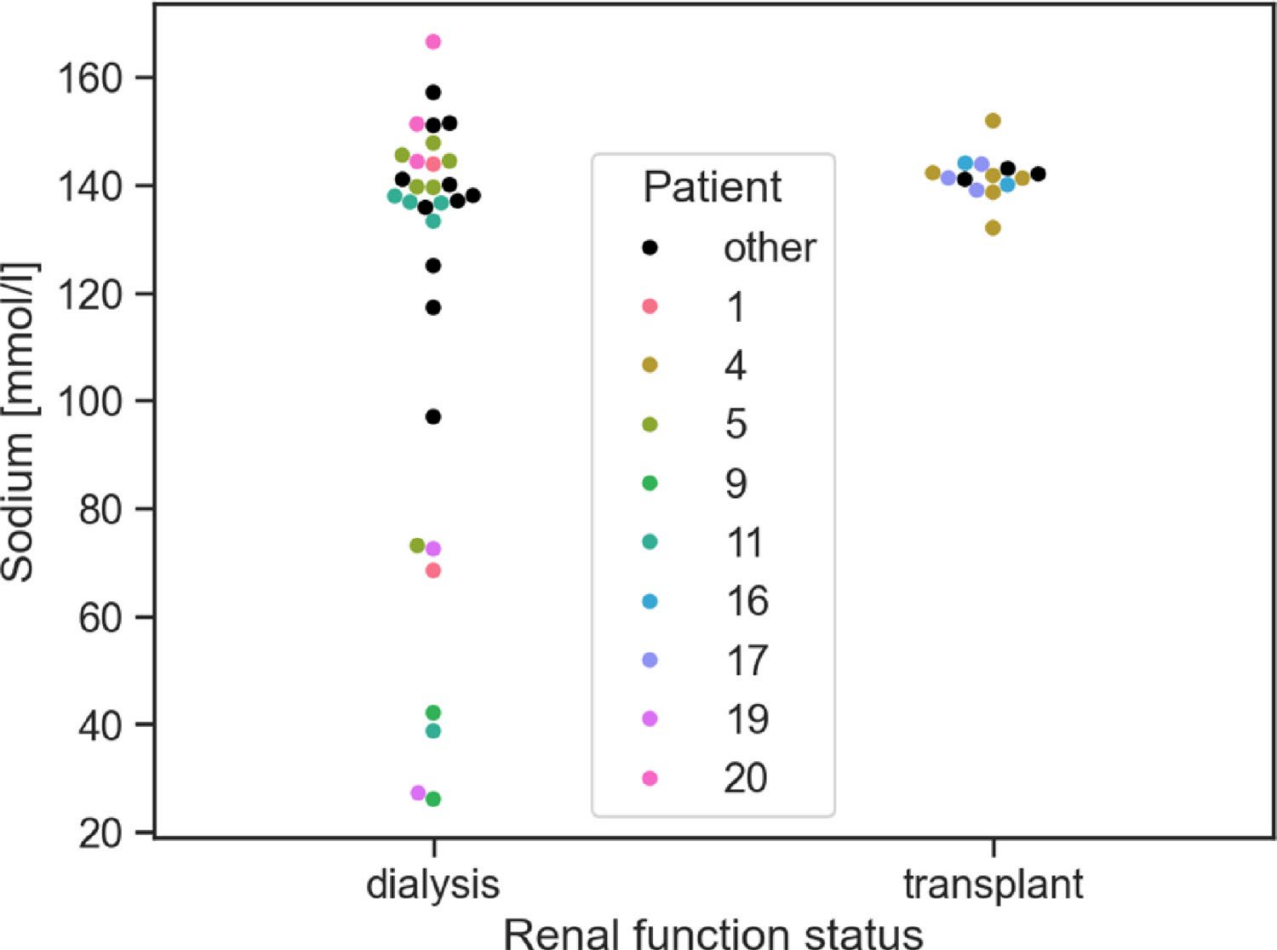
Plots of sodium concentration for cysts separated by the renal function status of the patients. The patients are marked with the same color as in Fig. 2

Sodium concentration was measured in 59 cyst fluid samples. 45 cysts from 23 patients yielded reliable measurements (see Fig. 3). Cysts were labeled as high-sodium (sodium ≥ 100 mmol/L) or low-sodium (sodium < 100 mmol/L). These labels are also shown in Fig. 1 (third column). Obviously, all low-sodium cysts were obtained from patients undergoing dialysis. Overall sodium values in this group ranged between 26.1 and 166.5 mmol/L. The transplant group featured only high-sodium cysts with concentrations between 132.0 and 151.9 mmol/l, i.e. within the physiological range for serum sodium.

**Fig. 4:**
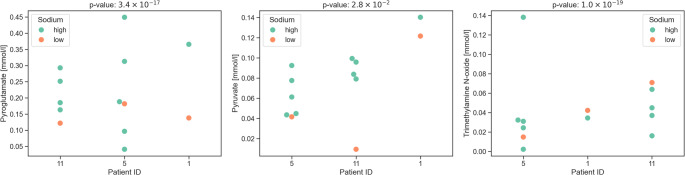
Plots of pyroglutamate, pyruvate, and trimethylamine N-oxide measured by NMR for cysts with different sodium concentrations. Cysts with sodium concentration ≥ 100 mmol/L are shown in green, and those with concentration < 100 mmol/L in orange. The patients are sorted by the respective intercept in the Mixed Linear Effects Model.

Additionally, we considered differences in cyst fluid composition based on the sodium level. A Mixed Linear Effects Model was used to estimate the difference by sodium level while accounting for inter-patient differences. Significant differences were observed for pyroglutamate (Bonferroni corrected p-value 3.4 × 10^− 17^), pyruvate (2.8 × 10^− 2^), and trimethylamine N-oxide (1.0 × 10^− 19^) (see Fig. 4).

### Total metabolite concentration of cyst fluid

Regarding the MS data, PQN (Probabilistic Quotient Normalization (Dieterle et al., 2006) factors were correlated with the sum of all features (TIC) (see Supplementary Fig. S3). A PQN factor larger than 1 implies a higher overall concentration in a sample in comparison to the median reference chromatogram, a PQN factors below 1 vice versa. Remarkably, the transplant group features significantly lower PQN factors (*p* = 3.67e^− 7^, Mann Whitney U test). Hence, the cyst fluids of this group revealed lower total metabolite concentrations on average. Furthermore, the PQN factors of the dialysis group scattered widely in comparison to the transplant group. So, renal cyst fluids of dialysis patients revealed higher differences in total metabolite concentrations.

## Discussion

The composition of renal cyst fluid has intrigued researchers since the initial studies conducted in the mid-20th century (Bricker & Patton, 1955; Lambert, 1947). Early investigations predominantly focused on electrolytes, creatinine, and urea (Gardner, 1969; Gardner et al., 1992; Huseman et al., 1980). Over the years, the complex nature of this biofluid has been examined from diverse perspectives (Foxall et al., 1992; Lai et al., 2008; Slade et al., 1998). To our knowledge, this study represents the first systematic metabolomic analysis of renal cyst fluid in an advanced-stage ADPKD cohort, comprising patients with ESKD that are either on dialysis (20 participants) or have received a kidney transplant previously (six participants). By leveraging high-resolution measurement technologies and advanced statistical approaches, we identified distinct metabolic signatures contingent on renal function status, thereby offering novel insights into the pathophysiology of ADPKD.

This study demonstrates a clear metabolic distinction between renal cyst fluid samples from dialysis patients and transplant recipients. Elevated levels of myoinositol, creatinine, sucrose, τ-methylhistidine, trigonelline, and sarcosine in dialysis patients are indicative of systemic metabolic derangements and altered cyst epithelial activity. In contrast, significantly increased concentrations of numerous proteinogenic amino acids in transplant recipients suggest restored metabolic stability and transformed conditions in native kidneys post-transplantation. Greater intra- and interindividual variability in the dialysis group compared to the more homogenous profiles observed in transplant recipients further underscores the systemic influences on cyst fluid composition and assumably the loss of original cyst epithelial features after transplantation. In both groups, fluid samples of different cysts from one patient were more similar than cyst fluids from different patients. Moreover, minimal impact of cyst volume on metabolic profiles was observed, with sarcosine as a rare exception driven by a single outlier. These findings not only highlight systemic and local factors driving cyst fluid composition but also emphasize its potential as a biomarker source for ADPKD management.

### ADPKD renal cyst fluid genesis

To answer, why kidney transplantation changes the metabolomic profile of ADPKD renal cyst fluid in such a profound way, the pathophysiology of renal cyst fluid genesis has to be clarified. As renal cysts are regarded to be anatomically separated from their parental tubule segment– except in the very early stage with cyst diameters under 2 mm– cyst fluid arises from a combination of plasma ultrafiltration and secretory activity of the cyst-lining epithelium (Paul & Vanden Heuvel, 2014; Sullivan et al., 1998; Terryn et al., 2011). Therefore, cyst fluid composition is regarded to depend on the biophysical and biochemical properties of the cyst epithelium. This comprises the quality of cell-to-cell contacts, the expression of ion channels and transporters as well as electrochemical gradients, which consequently will affect transport of water and solutes into the cyst lumen. In addition, the high metabolic activity of the cyst-lining cells (Bakaj & Pocai, 2023; Haumann et al., 2020) may impact the formation of metabolites that contribute to cyst fluid composition.

Previously, ADPKD renal cysts were classified according to their origin: “Proximal cysts” derived from the proximal nephron are characterized by leaky cyst walls permeable to many substances. “Distal cysts” derived from the distal nephron and collecting tubule reveal a tight epithelium able to evoke considerable gradients between plasma and cyst fluid, namely for sodium (low cyst/serum ratio), creatinine (high cyst/serum ratio) and numerous amino acids (high cyst/serum ratio) (Gardner, 1969; Gardner et al., 1992; Huseman et al., 1980).

### Metabolic signatures of renal cyst fluid

The clear dichotomy of the metabolomic profiles between dialysis patients and transplant recipients underscores the profound influence of renal function on cyst fluid composition.

Naturally, the physical status of both patient groups diverges dramatically. Differences in organ functions, renal replacement therapy itself, numerous drugs, and the nutritional status impact the organism (Tonelli et al., 2011). Moreover, renal transplantation changes the local conditions in the native ADPKD kidneys: immunosuppression, reduction in renal blood flow, abolished effect of uremia on tubular epithelium proliferation, and improvement of fluid overload may contribute to the significant decline in total kidney volume subsequent to transplantation (Jung et al., 2016; Veroux et al., 2018; Yamamoto et al., 2012). Both the altered systemic and intrarenal conditions will significantly affect the transepithelial secretion and metabolic activity of the cyst-lining epithelium.

#### Dialysis patients

In dialysis patients, the metabolomic profile of renal cyst fluid demonstrated considerable heterogeneity, likely attributable to the plentiful systemic factors associated with chronic renal failure and its management. These include comorbidities such as hypertension, malnutrition, and hyperparathyroidism, extensive use of medication, and the varying impact of long-term dialysis (Broseta et al., 2022; Stegmayr, 2017; Tonelli et al., 2011). Furthermore, the partially preserved variation of epithelial features depending on the cysts’ origin (Gardner, 1969; Gardner et al., 1992; Huseman et al., 1980) promoted the heterogeneity, which is especially showcased by varying sodium levels and probably by the widely scattered total metabolite concentrations.

Creatinine levels in cyst fluid were markedly elevated and heterogenous. On the one hand, this phenomenon reflects the impaired systemic clearance with oscillating high serum creatinine during intermittent dialysis treatment. On the other hand, the intraindividual variability in creatinine concentrations suggests residual heterogeneity of the cyst barrier function with the ability to accumulate creatinine in “distal cysts” (Gardner, 1969; Gardner et al., 1992; Huseman et al., 1980). Apparently, this feature is lost due to disease progression after transplantation. This hypothesis is underscored by the divergent variability of cyst creatinine and cyst sodium among dialysis and transplant patients.

Myoinositol, a well-established urinary biomarker of chronic kidney disease and, particularly, of ADPKD (Bevilacqua & Bizzarri, 2018; Dekker et al., 2022; Gronwald et al., 2011; Gil et al., 2018) was significantly elevated in the cyst fluids of dialysis patients compared to those of transplant recipients. In renal failure, not only urinary myoinositol rises but also serum myoinositol increases due to lower intrarenal enzymatic degradation (Bevilacqua & Bizzarri, 2018; Omosule et al., 2024). As cyst myoinositol of both groups significantly surpassed serum levels (Omosule et al., 2024), cyst-lining cells apparently accumulate and secrete myoinositol into the cyst lumina. The prominent gap between the dialysis and the transplant group might be primarily due to the renal function and the consecutively differing serum myoinositol (Meeusen et al., 2022).

Sucrose, the most abundant disaccharide found in plants, was uniquely abundant in the dialysis group, originating from medical interventions rather than dietary intake (Merino et al., 2019; Tasevska et al., 2005). For example, iron sucrose, commonly administered to address anemia in dialysis patients, may account for this observation. As human cells lack sucrose transporters, transepithelial transport relies on paracellular diffusion in a somewhat permeable cyst epithelium (Sullivan et al., 1998).

Three further molecules, namely τ-methylhistidine, trigonelline, and sarcosine, are significantly increased in the dialysis group. All three metabolites are discussed as novel serum biomarkers elevated in renal failure (Liu et al., 2024; Santos et al., 2023; Yamaguchi et al., 2021).

#### Transplant recipients

Allograft recipients, whose transplantations date back 12 to 60 months, displayed cyst fluid metabolomic profiles with significantly reduced inter- and interindividual variability reflecting the stabilization of systemic metabolic conditions and perhaps the progressive loss of typical cyst epithelial features post-transplantation (Jung et al., 2016; Torres et al., 2007b; Yamamoto et al., 2012). This assumption is supported by the consistently high cyst sodium concentrations similar to serum levels. However, the last observation might be biased by the low number of patients and cysts in the transplant group.

Elevated levels of proteinogenic amino acids, particularly leucine, isoleucine, valine, and alanine, were a hallmark of the transplant group. These findings suggest diminished intraepithelial amino acid turnover and metabolic demands after renal transplantation (Jung et al., 2016; Veroux et al., 2018; Yamamoto et al., 2012). Improved nutritional status post-transplantation will also affect cyst amino acid content (Małgorzewicz et al., 2008). Interestingly, mean concentrations of almost all detected amino acids exceed physiological serum levels in the transplant group, but not in the dialysis group. Glutamine represents the only amino acid with lower concentrations in cyst fluid than in serum in both the transplant and dialysis group. This may reflect the direct utilization of glutamine as carbon source by cyst cells (Foxall et al., 1992; Haumann et al., 2020).

The interplay between reduced renal perfusion, the missing pro-proliferative effects of uremia, and immunosuppressive therapy, particularly with calcineurin inhibitors such as tacrolimus, likely contributes to these metabolic changes (Jung et al., 2016; Marchetti & Navalesi, 2000; Oliveras et al., 2024; Shrestha, 2017; Torres et al., 2007b; Thölking et al., 2021; Veroux et al., 2018; Yamamoto et al., 2012). Tacrolimus-induced alterations of intrarenal amino acid pathways have been documented in both in vivo and in vitro models, suggesting a direct impact on cyst epithelial metabolism (Aouad et al., 2023; Xie et al., 2022). Since those analyses address healthy renal tubule cells, further research on the effects of immunosuppressants on ADPKD cyst fluid metabolomes is required, for example by leveraging our innovative 3D in vivo model, the chorioallantoic membrane model (Bichlmayer et al., 2022; Schueler et al., 2024).

Remarkably, drugs or their derivatives are able to pass the cyst epithelium. According to our MS data, renal cyst fluid of allograft recipients contained glucuronides of mycophenolic acid, thus metabolites of the immunosuppressant mycophenolate mofetil regularly administered in kidney allograft recipients. Transport of these metabolites requires active transport, with organic anion transporters facilitating their cellular uptake, while the multidrug resistance-associated protein 2 (MRP2) accounts for their efflux into the lumen of excretory organs including kidney (Patel et al., 2013).

### Limitations

Several limitations must be considered when interpreting this study’s results:

All participants suffered from advanced ADPKD with ESKD, either on dialysis or post-transplantation, which very likely do not reflect earlier disease. Furthermore, the study focused exclusively on patients undergoing nephrectomy, which might introduce selection bias, as nephrectomy is typically reserved for specific clinical indications (see Table 1). Though, only nephrectomy allows for systematic and ethical sampling of renal cysts.

The potential co-presence of acquired renal cysts, which frequently form in patients with long-term chronic kidney disease independently of ADPKD, may have introduced variability in the metabolomic profiles analyzed. Due to nephron loss for any reason, activated proto-oncogenes and growth factors lead to tubular hyperplasia and acquired cyst formation of the remaining healthy nephrons, exclusively the proximal tubules (Choyke, 2000; Rahbari-Oskoui & O’Neill, 2017). However, given the fact that ADPKD results in the formation of hundreds of cysts whereas acquired cysts are much lower in number, it is statistically unlikely that a significant number of acquired cysts have been included. Nevertheless, since acquired and hereditary cysts cannot be distinguished, we defined this point as a limitation.

Extrarenal factors such as comorbidities, renal replacement therapies, medication (see also Supplementary Table 2), and nutrition could not be fully controlled and may have influenced the results. We did not correct for these various possible confounders due to the limited cohort size.

The absence of direct plasma metabolomic data limits the ability to correlate systemic metabolic changes with cyst fluid composition. The lack of paired plasma and cyst fluid analysis may obscure the contribution of systemic versus local metabolic alterations.

Notably, the limited sample size, particularly in the transplant cohort, reduces the generalizability of the findings and warrants validation in larger studies.

With respect to assignments of NMR signals all significantly regulated metabolites were additionally verified by spike-in experiments and high resolution 2D spectra, while the assignment of non-significantly regulated metabolites was verified by 2D spectra alone.

### Clinical outlook and future research

The results of this study may have direct implications for clinical practice. The distinct metabolic signatures identified in renal cyst fluid underscore its potential as a diagnostic and prognostic biomarker in ADPKD. For instance, elevated myoinositol levels could aid in monitoring disease progression, while branched-chain amino acid profiles may provide insights into cyst epithelial activity and therapeutic responses. Moreover, the stabilization of metabolomic profiles post-transplantation may shed light on cystogenic pathways that may be addressed pharmaceutically in earlier stages of the disease. The analysis of cyst fluid of advanced stages of ADPKD may be regarded as a limitation. However, it may reflect the maximum amplitude of metabolic disorders in the course of ADPKD and, therefore, help to identify metabolic fingerprints that potentially already emerge at early stages of the disease and affect its progression. Therefore, we will measure those metabolites next in plasma and urine of early stage ADPKD patients and correlate the findings with renal function and cyst progression. Although our current data do not allow for causal correlations between cyst metabolome and cyst progression, the identified cyst fluid metabolites represent an excellent basis for further experiments to address the paracrine effects on cyst epithelium, e.g. using our already mentioned 3D in vivo model (Bichlmayer et al., 2022; Schueler et al., 2024). Additionally, exploring the impact of specific therapeutic agents, such as immunosuppressants, on cyst epithelial metabolism could provide novel insights into targeted treatment strategies. Lastly, leveraging advanced imaging techniques and machine learning approaches to correlate metabolomic data with clinical outcomes could pave the way for personalized medicine in ADPKD management, ultimately improving patient care and prognosis.

## Conclusion

This study provides a detailed characterization of renal cyst fluid metabolomics in advanced ADPKD, highlighting distinct metabolic signatures influenced by renal function status. These findings may not only enhance our understanding of ADPKD pathophysiology but also underscore the clinical potential of metabolomics in guiding disease management and therapeutic development. Future investigations should prioritize the integration of serum metabolomics and expand such analyses to earlier disease stages to elucidate the systemic and local interplay that drives cystogenesis.

## Electronic supplementary material

Below is the link to the electronic supplementary material.

Supplementary Material 1

Supplementary Material 2

## Data Availability

Raw data from NMR and MS (spectra and tables) will be available at MetaboLights (ID MTBLS12372). Additional data and the code used for analysis and creation of figures are available at https://git.uni-regensburg.de/behr-group-public/metabolomic-profiling-of-renal-cyst-fluid-in-advanced-adpkd.
