# Supplementary material for: Metabolomic profiling of renal cyst fluid in advanced ADPKD: insights from dialysis and transplantation cohorts
Source: Metabolomics. 2025 Jun 26;21(4):90. doi: 10.1007/s11306-025-02291-7 (PMC12202516; doi:10.1007/s11306-025-02291-7)
Supplement: Supplementary file 2 — Supplementary Material 2 [file 11306_2025_2291_MOESM2_ESM.docx]

**Supplementary**

**Methods: Liquid chromatography-mass spectrometry (LC-MS)**

For protein precipitation, 50 µL of cyst fluid were mixed with 200 µL pure methanol and 10 µL of a 0.1 mM internal standard mixture, containing ^13^C- and ^15^N-labelled or ^2^H-labelled amino acids. Then, samples were vortexed and stored overnight at -80 °C. The next day, samples were brought to room temperature, vortexed, centrifuged at 9,560 g and 4 °C for 5 min, and the supernatant was collected. To the remaining pellet 200 µL of 80% methanol were added. The samples were vortexed, centrifuged and the supernatant of the wash step was combined with first extract. This wash step was repeated once. The extracts were dried using a vacuum evaporator (CombiDancer, Hettich AG, Bäch, Switzerland), reconstituted in 100 µL H_2_O and transferred to LC-vial with glass inserts. As quality control, a pooled sample was generated using 5 µL of each extract. Furthermore, an extraction blank was prepared.

Untargeted metabolomics was performed using a Thermo Scientific Dionex Ultimate 3000 UPLC system (Idstein, Germany) coupled to a SCIEX Triple TOF 5600+ mass spectrometer (Sciex, Framingham, Massachusetts, USA). Chromatographic separation was carried out using an ACQUITY Premier HSS T3 reversed phase column with VanGuard FIT (Waters, 1.8 µm, 2.1 mm x 150 mm) and the respective pre-column. Eluent A was water with 0.1% formic acid (v/v) and eluent B was acetonitrile with 0.1% formic acid (v/v). A flow rate of 0.3 mL/min was used. Gradient elution with linear ramps was as follows: 3–50 % B in 8 min, 50–100% B in 1 min, 100% B for 3.5 min, 100-0% B in 1 min, and kept at 0% B for 8.5 min. The column was operated at 35 °C. Sample volumes of 5 µL were injected.

Ionization was performed using electrospray ionization in positive mode. The ESI source was kept at 400 °C and nitrogen was used as nebulizer (60 psi) and curtain gas (35 psi). The ion spray voltage was 5,000 V and information-dependent data acquisition (IDA) was employed. An IDA experiment consisted of a survey scan for 100 ms followed by 10 dependent MS/MS scans with an accumulation time of 50 ms each. The mass range was 50‑1000 m/z. Collision energy was set to 35 V, collision energy spread to ±15 V. The instrument was auto-calibrated by injecting a calibration sample every seventh injection. Samples were measured in a randomized order and the pooled sample, an in-house QC sample, and a blank sample were injected on a regular basis.

Raw data was converted into .mzml file format using the peak picking function in MSConvert (version 3.0.19, ProteoWizard, Palo Alto, CA, USA) with the parameter “vendor” (Chambers et al., 2012). Untargeted data processing with peak detection, deconvolution, peak alignment, and gap filling was performed with MZmine2 version 2.53 (Pluskal et al., 2010). The peak areas were extracted for further analysis. Features with more than 10 missing values and a relative standard deviation higher than 30% in the pooled samples (n=7) were removed. Furthermore, the average abundance of a feature in the pooled samples had to exceed the abundance in the extraction blank and the average abundance in water blanks (n=7) at least three times. Missing values in the filtered data set were imputed with one tenth of the peak area of the lowest peak area in the corresponding sample. Then, peak areas were normalized by Probabilistic Quotient Normalization (PQN) (Dieterle et al. 2006). Finally, a correlation filtering was performed, because analytes are often represented by multiple spectral features in the data set due to adduct formation, in-source fragmentation, multiple charge states, and di- or trimer ion formation. The highest abundant feature was kept from groups of features with the same retention time in a retention time window of 0.05 min, that were highly correlated across samples (R > 0.9). The pre-processed data set contained 1,190 features.

For feature annotation of significantly different features, spectral library search in MZmine (Version 4.5.20) (Pluskal et al. 2010)and LC-MS searches in the Human Metabolome Database (HMDB) (Wishart et al. 2022) were performed.

In MZmine, two different in-house compound/spectral libraries and public spectral libraries from three different sources were applied. Public libraries can be downloaded directly within MZmine.

| Public library | Source |
| --- | --- |
| Massbank of North America (MoNA) | <https://mona.fiehnlab.ucdavis.edu/> |
| Global Natural Products Social Molecular Networking (GNPS_All-No propagated) | https://external.gnps2.org/gnpslibrary |
| Zenodo (MS^2^ in positive mode) | https://zenodo.org/records/13911806 |

After alignment and preliminary annotation of features in MZmine, data was exported for further analysis in SIRIUS (Version 6.0.6) (Dührkop et al. 2019). Potential candidates identified through spectral library searches or HMDB searches were evaluated by formula/structure prediction in SIRIUS to confirm or reject the identification. Significantly different features between dialysis patients and the kidney-transplanted group with annotations are summarized in Supplementary Table 1.

Note, that the list of annotated features also contains entries for stable-isotope labelled amino acids. These were added to the samples in the beginning of sample preparation for quality control reasons. PQN corrects for global differences in concentration between samples by normalizing individual samples to a reference sample. Features in each sample are divided by a PQN-factor that is the median of the ratios of features in a sample chromatogram and the reference chromatogram. Thereby, with increasing deviation from this reference chromatogram the peak areas of individuals features are increased or reduced. As a result, this resulted peak areas for internal standard signals to be significantly different when testing between dialysis and the transplant group.

As expected, correlating the PQN factors with the sum of all features (TIC) results in a positive correlation (Figure S3). A PQN factor larger than 1 implies a higher overall concentration in a sample in comparison to the median reference, PQN factors below 1 indicate a more diluted sample compared to the median reference chromatogram. Interestingly, the transplant group features significantly lower PQN factors (p = 3.67e-7, Mann Whitney U test). Hence, the kidney cyst fluids of this group are more diluted on average.

**Additional Figures**


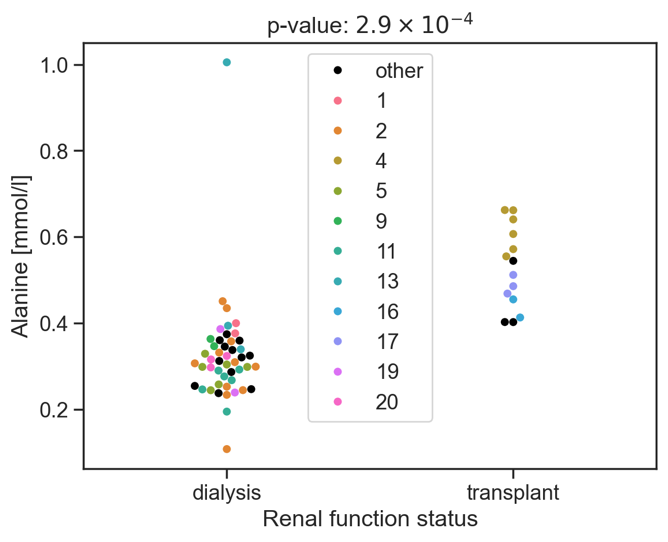

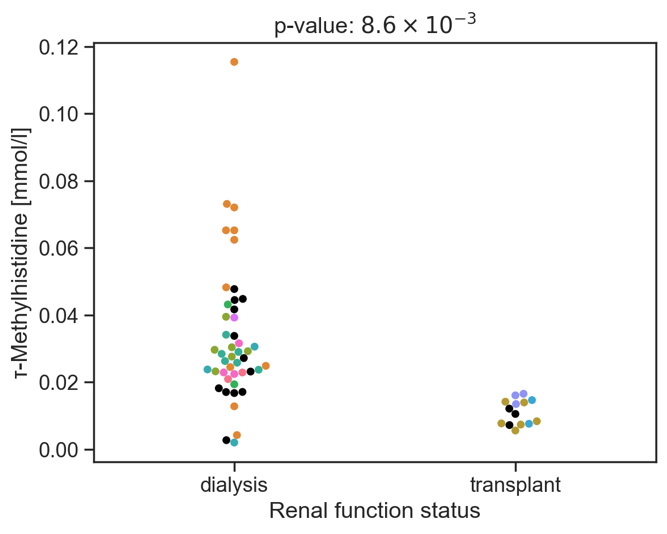

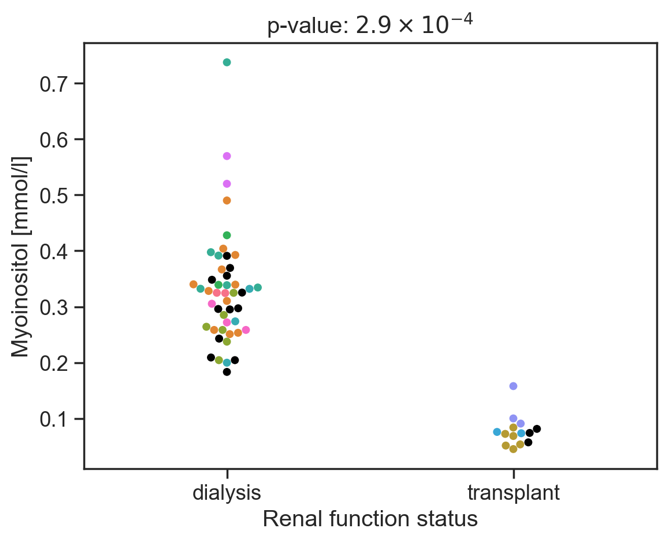

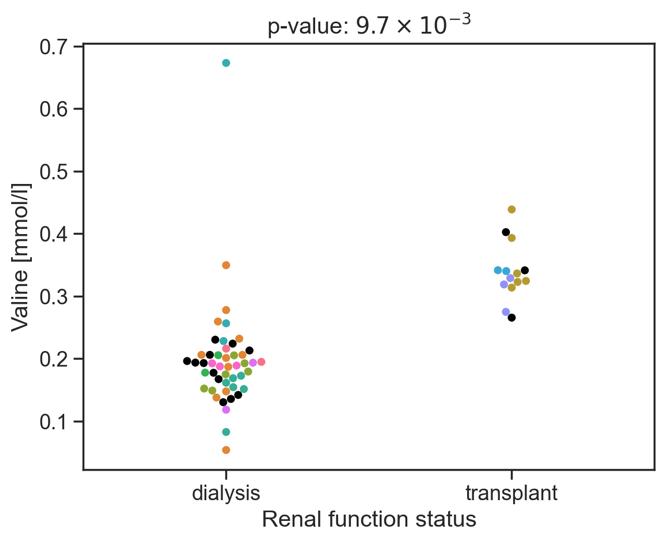

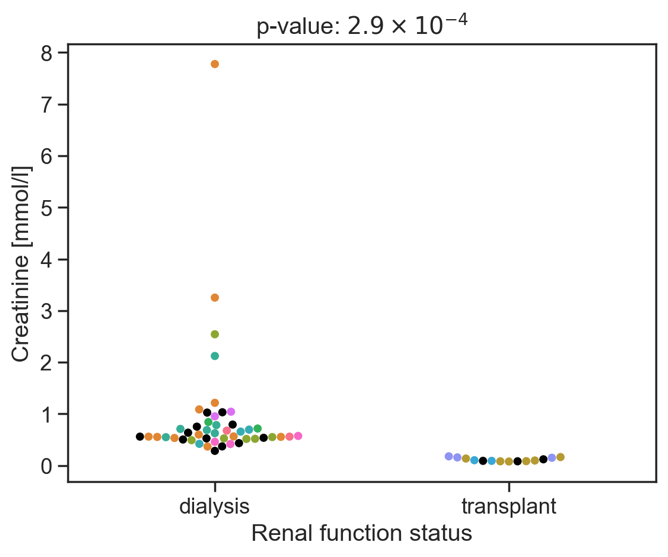

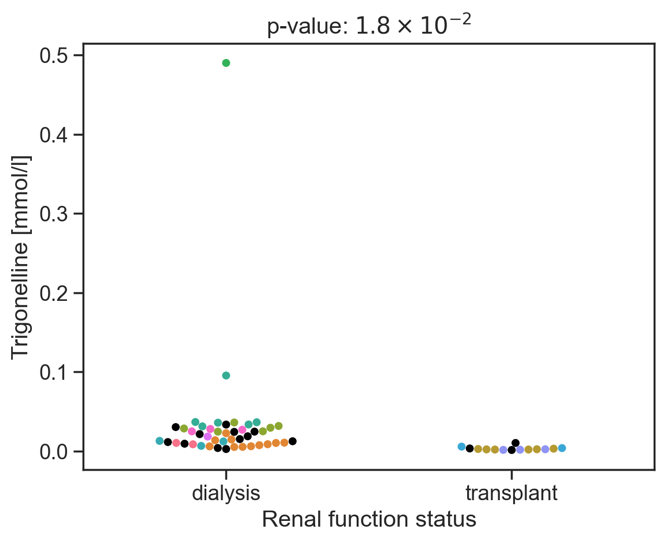

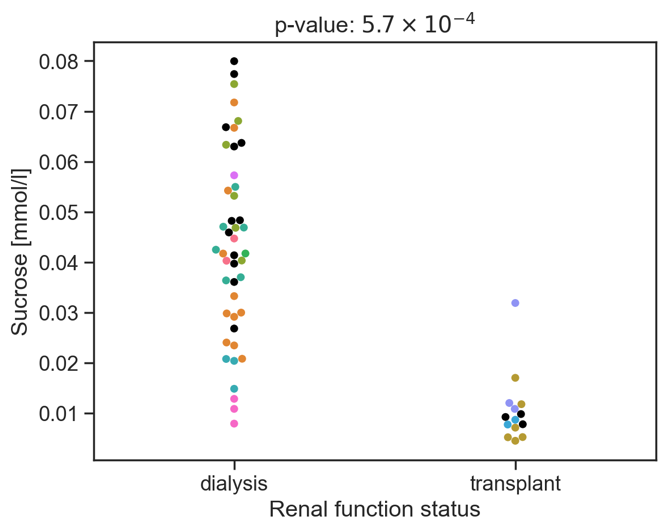

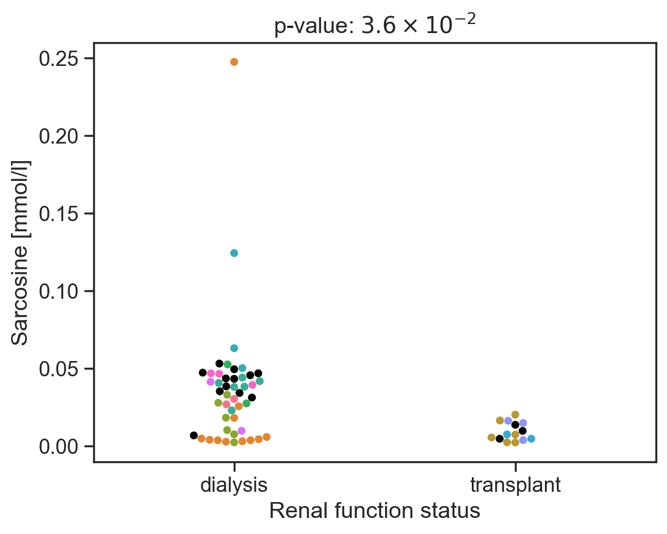

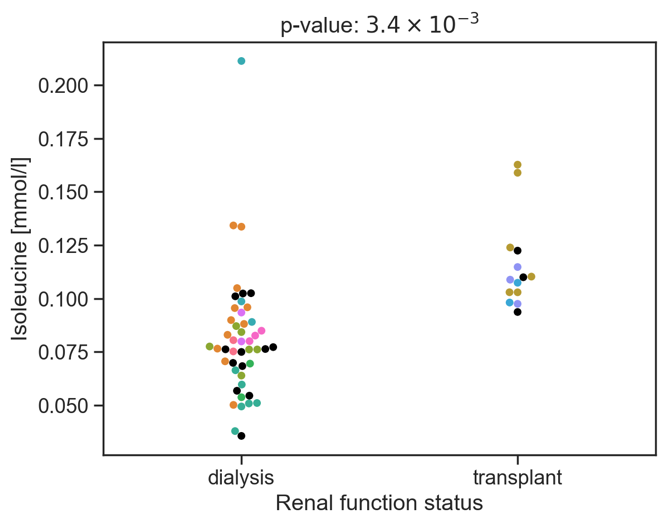

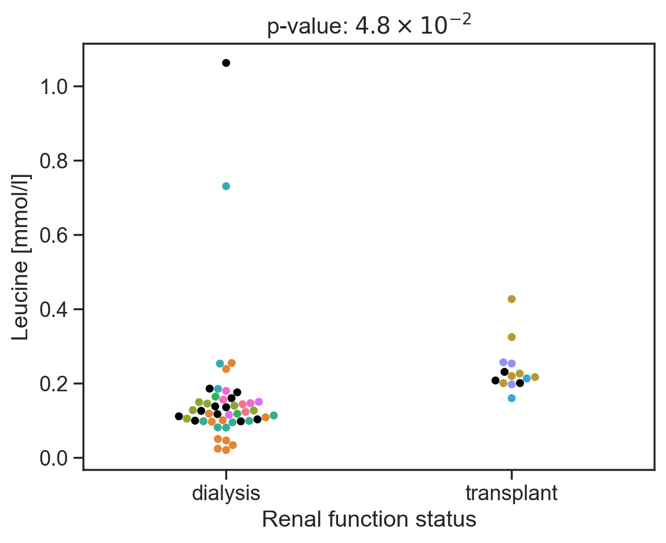


**Fig. S1:** Swarm plots for metabolites that differ significantly between dialysis and transplant group. The concentration (mM) is plotted.


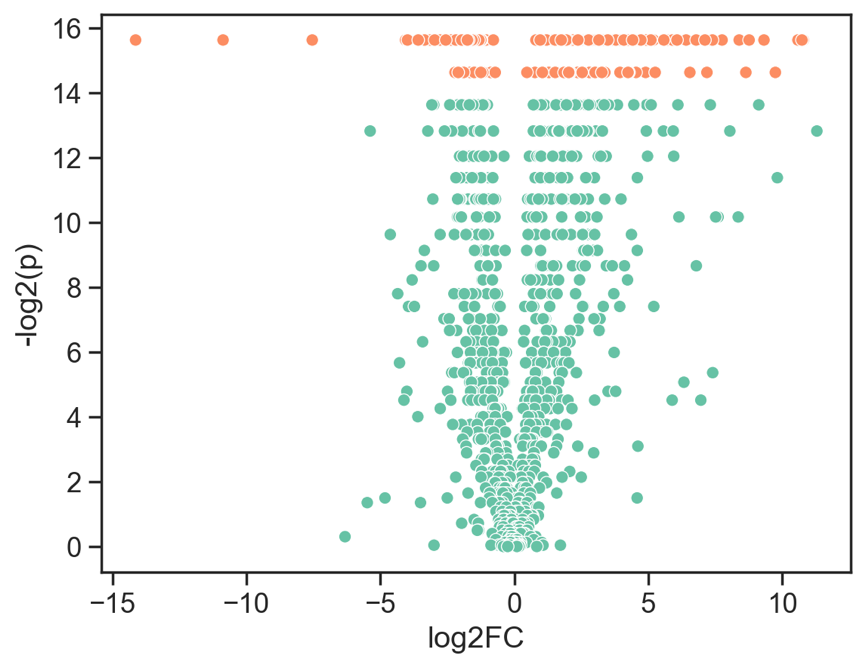


**Fig. S2:** Volcano plot for differences in MS data between dialysis and transplant group. The p-values are results of Mann-Whitney U rank tests, which can only return certain p-values. Differences which are significant after Bonferroni-correction are marked in orange. The fold change was calculated from the medians within the respective groups with a positive log2FC indicating a higher median in the dialysis group.


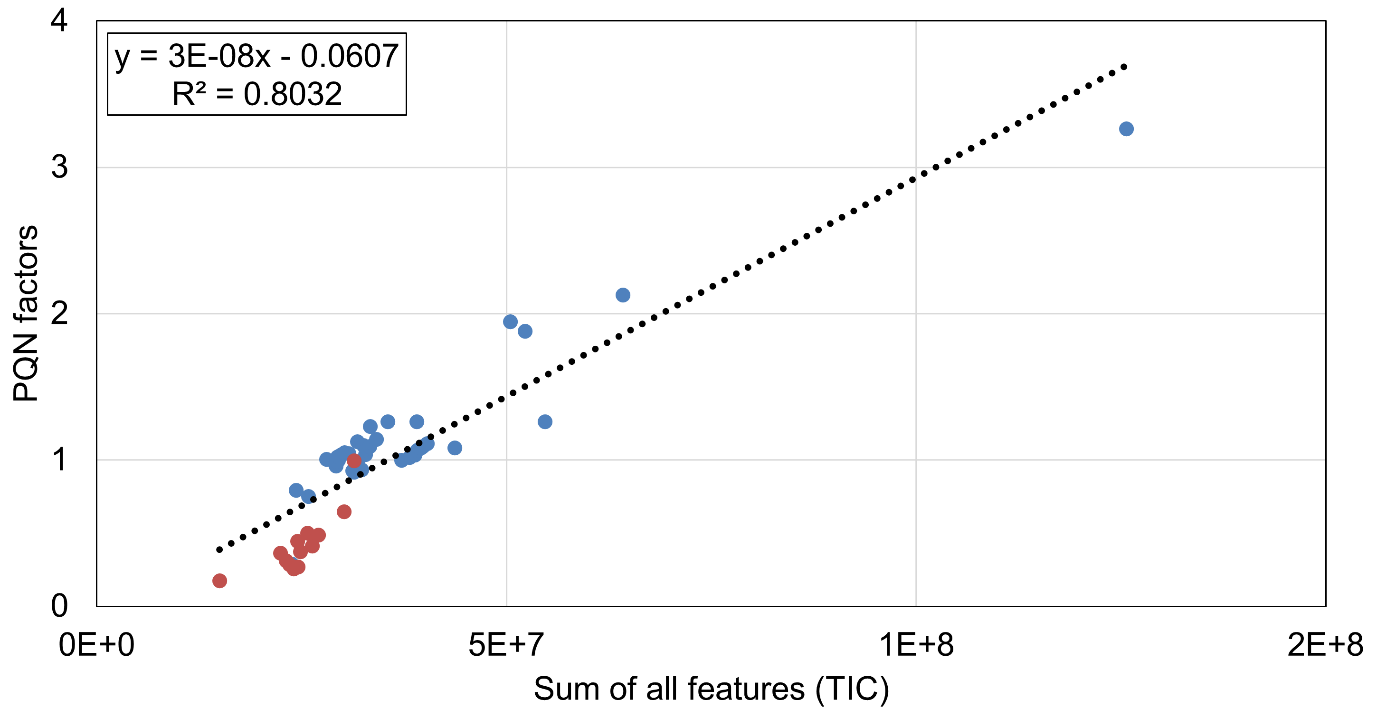


**Fig. S3:** Correlation of PQN factors with sum of all features (TIC). Renal function status is marked by blue (dialysis) and red (transplant).

**Supplementary Table 1** (Excel file): List of significant features from MS data distinguishing dialysis and transplant patient.

|  | **All** | **Dialysis** | **Transplant** | **p-value** |
| --- | --- | --- | --- | --- |
| **N (percentage)** | **26 (100%)** | **20 (76.9%)** | **6 (23.1%)** | • |
| antihypertensive drugs | 24 (92.3%) | 19 (95.0%) | 5 (83.3%) | 0.4154 |
| diuretics | 12 (46.2%) | 11 (55.0%) | 1 (16.7%) | 0.1696 |
| cholesterol-lowering drugs | 10 (38.5%) | 5 (25.0%) | 5 (83.3%) | 0.0184 |
| anticoagulants | 3 (11.5%) | 2 (10.0%) | 1 (16.7%) | 1.0000 |
| platelet aggregation inhibitors | 6 (23.1%) | 4 (20.0%) | 2 (33.3%) | 0.5960 |
| phosphate binders | 15 (57.7%) | 15 (75.0%) | 0 (0.0%) | 0.0020 |
| bicarbonate | 15 (57.7%) | 10 (50.0%) | 5 (83.3%) | 0.1973 |
| calcimimetic agents | 5 (19.2%) | 5 (25.0%) | 0 (0.0%) | 0.2981 |
| antidiabetics | 4 (15.4%) | 3 (15.0%) | 1 (16.7%) | 1.0000 |
| vitamin D | 19 (73.1%) | 14 (70.0%) | 5 (83.3%) | 1.0000 |
| dietary supplement, other vitamins | 15 (57.7%) | 14 (70.0%) | 1 (16.7%) | 0.0538 |
| anti-gout drugs | 2 (7.7%) | 2 (10.0%) | 0 (0.0%) | 1.0000 |
| immunosuppressive agents | 7 (26.9%) | 1 (5.0%) | 6 (100.0%) | 0.0000 |
| - corticosteroids | 5 (19.2%) | 1 (5.0%) | 4 (66.7%) | 0.0047 |
| - calcineurin inhibitors | 6 (23.1%) | 0 (0.0%) | 6 (100.0%) | 0.0000 |
| - mycophenolate mofetil | 6 (23.1%) | 0 (0.0%) | 6 (100.0%) | 0.0000 |

**Supplementary Table 2**: Medication of the ADPKD cohort. Given are the absolute number and percentage of patients who received the medication at time of nephrectomy, as well as the p-value of Fisher’s exact test.

**References**

Dieterle, F., Ross, A., Schlotterbeck, G., & Senn, H. (2006). Probabilistic quotient normalization as robust method to account for dilution of complex biological mixtures. Application in 1H NMR metabonomics. *Anal Chem, 78*, 4281–4290. doi:10.1021/ac051632c.

Dührkop, K., Fleischauer, M., Ludwig, M., Aksenov, A. A., Melnik, A. V., Meusel, M., et al. (2019). SIRIUS 4: a rapid tool for turning tandem mass spectra into metabolite structure information. *Nat Methods, 16*, 299–302. doi:10.1038/s41592-019-0344-8.

Pluskal, T., Castillo, S., Villar-Briones, A., & Oresic, M. (2010). MZmine 2: modular framework for processing, visualizing, and analyzing mass spectrometry-based molecular profile data. *BMC Bioinformatics, 11*, 395. doi:10.1186/1471-2105-11-395.

Wishart, D. S., Guo, A., Oler, E., Wang, F., Anjum, A., Peters, H., et al. (2022). HMDB 5.0: the Human Metabolome Database for 2022. *Nucleic acids research, 50*, D622-D631. doi:10.1093/nar/gkab1062.
